# Simultaneous Anterior Glenohumeral Dislocation and Ipsilateral Acromioclavicular Separation: A Dual Injury of the Shoulder

**DOI:** 10.7759/cureus.1582

**Published:** 2017-08-19

**Authors:** Ömer Faruk Kılıçaslan, Baver Acar, Aziz Atik, Ozkan Kose

**Affiliations:** 1 Department of Orthopaedics and Traumatology, University of Health Sciences, Medical Faculty, Antalya Education and Research Hospital, Antalya, Turkey

**Keywords:** dislocation, shoulder, anterior, acromioclavicular, separation

## Abstract

Isolated acromioclavicular separations or shoulder dislocations are common injuries. However, a combination of complete acromioclavicular separation and anterior shoulder dislocation is extremely rare. Herein we present a combination of anterior shoulder dislocation and type III acromioclavicular separation that was succesfully treated conservatively. Orthopaedic surgeons should have a high clinical suspicion in daily practice. We believe that both pathologies can be treated conservatively.

## Introduction

The glenohumeral joint is the most mobile and therefore the most frequently dislocated joint in the human body. These dislocations represent an estimated incidence rate of 23.9 (95% confidence interval, 20.8-27.0) per 100,000 persons/year [1]. Acromioclavicular (AC) injuries are less common than shoulder dislocations. Injuries to the acromioclavicular joint account for approximately 12% of those to the shoulder girdle in clinical practice [2]. Simultaneous dislocation of both joints is an extremely rare issue. Isolated shoulder dislocations and isolated acromioclavicular separations are two common injuries of the shoulder girdle, which are usually seen in young active subjects. However, simultaneous ipsilateral glenohumeral and acromioclavicular dislocation is extremely rare, and to the best of our knowledge, this injury has not been reported in current literature. Diaphyseal fractures of the clavicle can be in association with an injury to the acromioclavicular joint, and a separated injury may be difficult to diagnose, particularly with marked displacement of the clavicular fracture. Less commonly, a complete separation of the clavicle, the ‘floating clavicle,’ with dislocation of both acromioclavicular and sternoclavicular joints may present [3]. To the best of our knowledge, this “acromioclavicular separation and anterior shoulder dislocation” injury pattern has not been reported in the literature before.

## Case presentation

**Figure 1:**
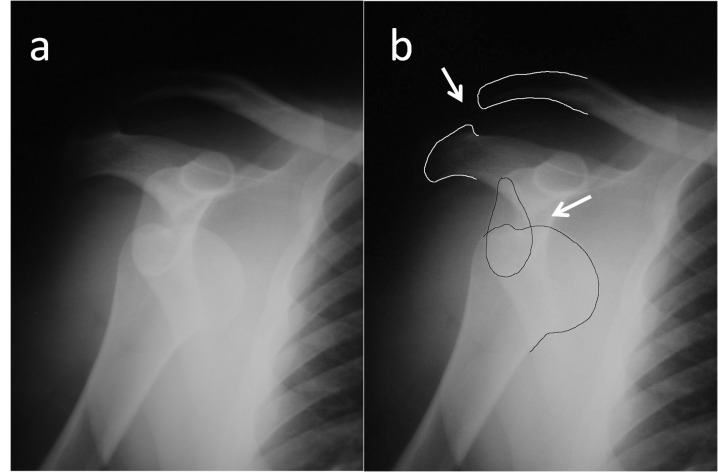
True AP (a) and illustrated (b) roentgenographies showing both anterior glenohumeral joint dislocation and acromioclavicular type III separation AP - anteroposterior

**Figure 2:**
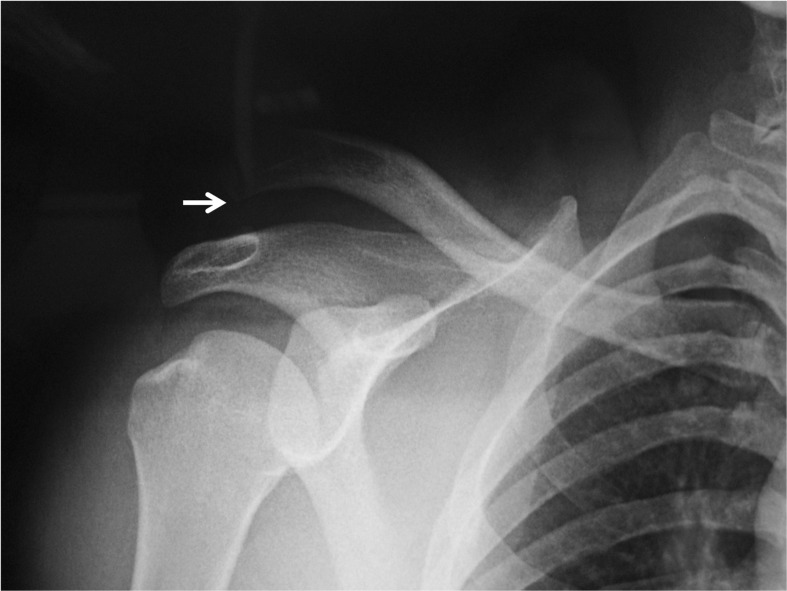
Radiological view after conservative treatment

A 35-year-old male patient was admitted to our emergency department after sustaining a motorcycle accident. He was conscious and oriented and his vital signs were normal at initial admission. His major complaint was right shoulder pain and deformity. On physical examination, both epaulet and step-off signs were seen on his right shoulder. Active shoulder motion was absent and passive motions were extremely painful in every direction. The neurovascular examination was otherwise normal. A direct radiographic examination revealed simultaneous dislocation of the shoulder and acromioclavicular joints (Figure 1). The glenohumeral joint was reduced under sedation with the Kocher maneuver, and a control radiograph showed the proper reduction of the shoulder joint and complete type III AC separation (Figure 2). After obtaining an explanation of the benefits and risks of surgical treatment on type III AC dislocation, the patient chose conservative treatment. The patient wore a Velpeau bandage for two weeks; thereafter, active pendulum exercises were started and gradually increased to full range of shoulder movements. At fifth week, the patient returned to his previous job (accountant). At the final follow-up two years after the initial injury, the patient was free of pain with normal shoulder movements without a history of redislocation. However, he complained about the appearance of his right shoulder and prominence over the AC joint.

## Discussion

Anterior shoulder dislocations account for over 95% of all shoulder dislocations. Rouleau, et al. reported a review of the literature with 477 patients (547 shoulders) with 90 of them being women (19%). The average age of these patients was 41.1 years (range 17–75 years). In 70 cases, the dislocations were bilateral. A majority of dislocations (65%) had associated injuries: fracture was the most common followed by reverse Hill-Sachs deformities and cuff tears [4]. The arm is usually in an abducted and externally rotated position. There is absence of the normal contour of the deltoid and the acromion is prominent posteriorly and laterally. The humeral head itself may well be palpable anteriorly. It is easy to identify anterior shoulder dislocations with plain anteroposterior (AP) radiographs. In any case of suspicion, scapula Y and other shoulder AP radiographies should be taken. The axillary view greatly assists in making the diagnosis. Computerized tomography (CT) may have a role in depicting comorbid pathologies. Magnetic resonance imaging (MRI) can detect anterior capsulolabral complex and rotator cuff injuries. Treatment of an anterior shoulder dislocation depends on the dislocated time period, reducibility, and comorbid pathologies. The management after reduction traditionally involves immobilisation in internal rotation. The typical length of immobilisation is three to six weeks, but there is a trend toward shorter periods or no immobilisation at all.

Traumatic acromioclavicular joint separations are common injuries among the active population [5]. The mechanism of most acromioclavicular separations is trauma, caused either by a direct blow to the top of the shoulder with the arm in the adducted position or by a fall on an outstretched hand. Rockwood classified acromioclavicular separations into six types [6]. In type I injuries there is partial and in type II complete disruption of the acromioclavicular ligaments. In both, the radiographs will appear to be normal. In type III injuries, the vertical translation at the joint is up to the width of the clavicle while in type IV the clavicle is displaced posteriorly into the trapezius. In type V injuries, the degree of separation is greater because of the concomitant disruption of the deltotrapezius fascia attached to the end of the clavicle. In the type VI injury the clavicle is displaced inferiorly and comes to lie below the coracoid process underneath the conjoint tendon.

Conservative treatment is almost universally applicable to type I and type II injuries. The most common form of non-operative treatment involves simple analgesia, topical ice therapy and rest in a sling to give relief from symptoms. The treatment of isolated type III acromioclavicular separations is controversial. While some authors advise surgery, succesful results were also reported by conservative means. The initial treatment of acute type III acromioclavicular separations is usually conservative, although some authors do advocate surgery for acute injuries in high performance throwing athletes. However, the current view remains in favour of conservative management of acute type III injuries. Operation is used to treat medically-fit patients with type IV and type V injuries. Gstettner, et al. conducted a retrospective study in which 24 patients who were treated surgically with a hook plate and 17 patients who were treated conservatively were examined with a mean follow-up of 34 months. In this study, better results were achieved by surgical treatment with the hook plate than by conservative treatment [7]. Smith, et al. reported their meta-analysis, which is about operative versus non-operative management following type III acromioclavicular dislocation. There was no statistically significant difference between the interventions in respect to strength, pain, throwing ability, loss of anatomical reduction, ossification of the coracoclavicular ligament, or acromioclavicular joint osteoarthritis. Non-operative management was associated with significantly poorer cosmetic outcome but less sick leave compared to operative management. Also, they found significantly higher risk of infection following surgical management compared to non-operative treatment [8].

Dias, et al. reviewed 44 patients in the fifth year after conservative treatment for acromioclavicular dislocation, and of these, 42 had a good result and two had fair outcomes [9]. Acromioclavicular dislocation may contribute to other shoulder girdle traumas. Tischer, et al. reported that an incidence of intra-articular injuries were found in 14 of 77 patients (18.2%) with acute acromioclavicular joint dislocations, and mostly superior labral anterior posterior (SLAP) lesions were observed in 11 of 77 patients (14.3%) [5]. Perez, et al. reported a case who had an acromioclavicular dislocation type III with a distal clavicle fracture type I in the same arm [10]. At least one fracture is advised to be fixed for shoulder multitraumas and especially for floating shoulders. But we succesfully treated our patient conservatively. No sign of instability or redislocation happened during the two-year follow-up period.

## Conclusions

Anterior shoulder dislocation and accompanying type III acromioclavicular separation has not been reported in the literature before. It is obvious that orthopaedic surgeons should have a high clinical suspicion in daily practice. We believe that both pathologies can be treated conservatively.
